# Fasted intestinal solubility limits and distributions applied to the biopharmaceutics and developability classification systems

**DOI:** 10.1016/j.ejpb.2021.12.006

**Published:** 2022-01

**Authors:** Qamar Abuhassan, Ibrahim Khadra, Kate Pyper, Patrick Augustijns, Joachim Brouwers, Gavin W. Halbert

**Affiliations:** a Strathclyde Institute of Pharmacy and Biomedical Sciences, University of Strathclyde, 161 Cathedral Street, Glasgow G4 0RE, United Kingdom; b Department of Mathematics and Statistics, University of Strathclyde, Livingstone Tower, 26 Richmond Street, Glasgow G1 1XH, United Kingdom; c Drug Delivery and Disposition, KU Leuven, ON2, Herestraat 49 Box 921, 3000 Leuven, Belgium

**Keywords:** Solubility, Fasted simulated intestinal fluid, Biopharmaceutics classification system, Developability classification system, Ibuprofen, Mefenamic acid, Griseofulvin, Dipyridamole, Furosemide, Paracetamol, Acyclovir

## Abstract

After oral administration, a drug’s solubility in intestinal fluid is an important parameter influencing bioavailability and if the value is known it can be applied to estimate multiple biopharmaceutical parameters including the solubility limited absorbable dose. Current in vitro measurements may utilise fasted human intestinal fluid (HIF) or simulated intestinal fluid (SIF) to provide an intestinal solubility value. This single point value is limited since its position in relation to the fasted intestinal solubility envelope is unknown. In this study we have applied a nine point fasted equilibrium solubility determination in SIF, based on a multi-dimensional analysis of fasted human intestinal fluid composition, to seven drugs that were previously utilised to investigate the developability classification system (ibuprofen, mefenamic acid, furosemide, dipyridamole, griseofulvin, paracetamol and acyclovir). The resulting fasted equilibrium solubility envelope encompasses literature solubility values in both HIF and SIF indicating that it measures the same solubility space as current approaches with solubility behaviour consistent with previous SIF design of experiment studies. In addition, it identifies that three drugs (griseofulvin, paracetamol and acyclovir) have a very narrow solubility range, a feature that single point solubility approaches would miss. The measured mid-point solubility value is statistically equivalent to the value determined with the original fasted simulated intestinal fluid recipe, further indicating similarity and that existing literature results could be utilised as a direct comparison. Since the multi-dimensional approach covered greater than ninety percent of the variability in fasted intestinal fluid composition, the measured maximum and minimum equilibrium solubility values should represent the extremes of fasted intestinal solubility and provide a range. The seven drugs all display different solubility ranges and behaviours, a result also consistent with previous studies. The dose/solubility ratio for each measurement point can be plotted using the developability classification system to highlight individual drug behaviours. The lowest solubility represents a worst-case scenario which may be useful in risk-based quality by design biopharmaceutical calculations than the mid-point value. The method also permits a dose/solubility ratio frequency distribution determination for the solubility envelope which permits further risk-based refinement, especially where the drug crosses a classification boundary. This novel approach therefore provides greater in vitro detail with respect to possible biopharmaceutical performance in vivo and an improved ability to apply risk-based analysis to biopharmaceutical performance. Further studies will be required to expand the number of drugs measured and link the in vitro measurements to in vivo results.

## Graphical abstract

**Figure:**
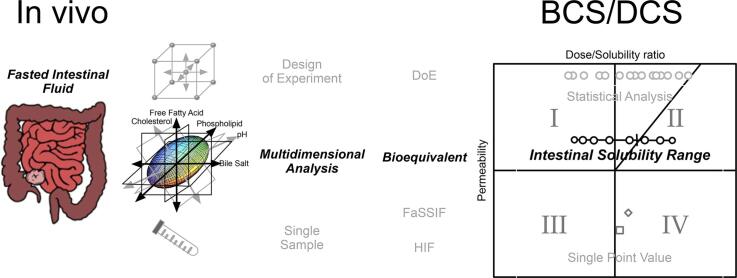

## 1 Introduction

### 1.1 Oral drug administration

The oral route is the most common method of drug administration. It permits self-administration, which provides patient acceptability, assists compliance and allows the pharmaceutical industry to meet this demand through the provision of adaptable and stable solid oral dosage forms. The apparent simplicity of this approach, however, hides a complexity arising from the combination of gastro-intestinal tract anatomy and physiology along with the physicochemical properties of the administered drug and dosage form. It has been recognised [1] that amongst the factors controlling drug absorption is the drug’s solubility in intestinal fluid since solid drug particles are not absorbed. This was formalised in the Biopharmaceutics Classification System [2], that linked solubility and permeability with in vitro and in vivo performance, with applicability to regulatory situations covering oral products especially around solubility and dissolution.

### 1.2 Intestinal solubility calculations

Solubility as a factor in oral absorption permitted, by using a range of assumptions, the development of drug absorption models that could be applied to calculate or estimate the absorption of drugs. One of the first values proposed was the Absorption Potential (AP, Eq. (1)) [1],

(1) $$\mathrm{AP}=\log\left(P\times F_{\mathrm{non}}\times \frac{S_{0}V_{L}}{X_{o}}\right)$$

where P is effective gut wall permeability to the drug, F_non_ is the fraction non-ionised at pH 6.5, S_0_ is the intrinsic solubility (aqueous solubility of the non-ionised species at 37 °C), V_L_ is the small intestinal water volume (mL), and X_o_ is the dose administered. A further variation is the Maximum Absorbable Dose (MAD), which could be calculated using Eqs. (2) [3] and (3) [4];

(2) $$\mathrm{MAD}=S\times K_{a}\times SIWV\times SITT$$

(3) $$\mathrm{MAD}=P_{\mathrm{eff},human}\times S\times A\times T_{\mathrm{si}}$$

where, S is the solubility at pH 6.5, K_a_ is the transintestinal absorption rate constant (min^−1^), SIWV is the small intestinal water volume (mL), SITT and T_si_ are the small intestinal transit time (min), P_eff human_ is human jejunal drug permeability (cm s^−1^), and A is the absorption surface area (7.54 × 10^4^ cm^2^). These equations utilise aqueous solubility at pH 6.5, however, it was recognised that aqueous solubility is not identical to intestinal solubility [5] due to the presence of solubilising agents such as bile salts and phospholipids. This approach was further modified with the dimensionless Dose Number (D_o_, Eq. (4)) [6];

(4) $$D_{o}=\frac{D/V_{0}}{S}$$

where D is the dose administered, V_o_ is the volume of water taken and S is the physiological solubility. This introduces the dose/solubility ratio concept, which is further expanded in the Developability Classification System (DCS) [7] and also required the use of a “physiological” solubility value rather than a simple aqueous value. This led to the Solubility Limited Absorbable Dose (SLAD, Eq. (5)) [7], [8];

(5) $$\mathrm{SLAD}=S_{\mathrm{si}}\times V\times M_{p}$$

where S_si_ is the estimated small intestinal solubility (mg mL^−1^), V is the volume of fluid (500 mL) and M_p_ is the permeability dependent multiplier. For a high permeability drug M_p_ is equal to the absorption number (A_n_, Eq. (6)); for low permeability drugs is set equal to 1. The A_n_ is defined as the ratio between the mean small intestinal transit time (T_si_ 3.32 h) to absorption time (R/P_eff_), where R is the intestinal radius (1.25 cm [6]) and P_eff_ the effective permeability of the intestine to the drug.

(6) $$A_{n}=\frac{P_{\mathrm{eff}}\times T_{\mathrm{si}}}{R}$$

The solubility value required to calculate SLAD is the intestinal equilibrium solubility [7], which can be measured in intestinal fluid or simulated intestinal fluids. A recent refinement of the DCS [8] proposes standardisation of the solubility criteria with the use of fasted human intestinal fluid (HIF) as a “gold standard” approach, since this is the most biorelevant. However, the authors recognise that “fasted HIF samples are difficult to handle and quite expensive” and that a surrogate of fasted simulated intestinal fluid (SIF) “is an attractive alternative”. The recommendation proposed using solubility values in either fasted HIF or fasted SIF and a correlation between the two systems, based on literature results, is presented.

### 1.3 Intestinal solubility measurement

Equilibrium solubility measured in aspirates of fasted HIF is known to be variable [9], [10], as is the composition of HIF aspirates [11]. The application of fasted HIF as a solubility determination medium is also restricted, as discussed in the refined DCS [8], by the limited volumes extracted during sampling and the intrusive nature of the sampling process [12]. Multiple fasted SIF recipes have been developed to overcome HIF limitations [13], [14] and a correlation between drug solubility in HIF and SIF [8], [15] can be determined. Despite this relationship, it is not evident which SIF recipe is optimal [13], new recipes are still in development [16] and the measured solubility for drugs varies between recipes [16] and measurements [9]. Recent design of experiment (DoE) guided studies of the impact of SIF media components on drug solubilisation [14], [17], [18], [19] highlight the variation and complexity that is inherent within these SIF media systems. In order to unify the various approaches, a recent study performed a multi-dimensional analysis of an extensive fasted HIF chemical compositional data set [11] to calculate eight points or HIF compositions [20] that provided a greater than ninety percent coverage of the compositional space in five dimensions. Along with a central distribution point, the nine compositions have been applied as a set of fasted SIF media recipes to explore equilibrium intestinal solubility [21]. The resulting solubility distributions are statistically equivalent to the previous DoE guided studies of the fasted state [14], [17], [22], and encompass published solubility values in either fasted HIF samples or SIF recipes. This recent result indicates that intestinal solubility is a range and not a single value. Application of a single solubility value measured either in fasted HIF or SIF in the calculations detailed above will therefore represent a mid-point and will not provide information on the potential range or distribution of the solubility due to the inherent variability of intestinal conditions that influence solubility.

### 1.4 Intestinal solubility and developability classification system

In this study, we have applied the fasted intestinal fluid media compositions identified using the multi-dimensional analysis [20] to the drugs (excluding digoxin) assessed in the original Developability Classification System [7] for the fasted state. The equilibrium solubility of ibuprofen, mefenamic acid, furosemide, dipyridamole, griseofulvin, paracetamol, and acyclovir, has been determined in the nine media recipes [20], [21], along with a value in simulated fasted simulated intestinal fluid (FaSSIFv1) version 1 [14]. The nine media recipes provide a range of solubility values that, due to the derivation from sampled HIF, covers the fasted HIF range and can therefore be considered bioequivalent. The solubility values therefore can be applied to the DCS grid and associated calculations that predict absorption to provide the limits for likely in vivo solubility behaviour. Finally, a solubility frequency distribution within those limits can be determined to assess solubility behaviour across the population range, based on the twenty volunteers sampled in the original study [11]. It should be noted that the frequency distribution represents the aggregated measured HIF compositions from all volunteers and therefore intra- and inter-subject variability cannot be analysed using this approach.

## 2 Materials and methods

### 2.1 Materials

Sodium taurocholate, cholesterol, sodium chloride (NaCl), sodium oleate, ammonium formate, formic acid, potassium hydroxide (KOH), hydrochloric acid (HCl), griseofulvin, furosemide, dipyridamole, and acyclovir were purchased from Merck Chemicals Ltd. Ibuprofen was obtained from BSAF chemical company, Paracetamol was from Mallinckrodt Pharmaceuticals and mefenamic acid from Sigma Aldrich. Phosphatidylcholine from soybean (PC S) was purchased from Lipoid company. See Table 1 for physicochemical properties and molecular structures. Chloroform was from Rathburn Chemical Company, FaSSIF media was purchased from Biorelevant.com, and sodium phosphate monobasic monohydrate (NaH_2_PO_4_·H_2_O) was purchased from Fisher Scientific. All acetonitrile (ACN) and methanol (MeOH) solvents were HPLC gradient (VWR). All water is ultrapure Milli-Q water.

**Table 1:** Physicochemical properties and molecular structures of drugs.

Compound	a/b/n	pKa	LogP	Structure
Ibuprofen	a	5.3	3.97	

Mefenamic Acid	a	4.2	5.12	

Furosemide	a	3.9	2.03	

Dipyridamole	b	6.2	3.77	

Paracetamol	n	–	0.46	

Griseofulvin	n	–	2.18	

Acyclovir	n	2.52/9.35	−1.56	

### 2.2 Methods

#### 2.2.1 Solubility media preparation

##### 2.2.1.1 Bioequivalent media stock solutions

For each media recipe (Table 2), a concentrated lipid stock was prepared as follows. The required (×15) weight of bile salt (sodium taurocholate), phospholipid (soyabean lecithin) and free fatty acid (sodium oleate) for each media recipe was dissolved in chloroform (3 mL) – Stock A. The required weight of cholesterol (×1500) for each media recipe was dissolved in chloroform (10 mL) – Stock B. An aliquot of Stock B (0.1 mL) was added to each Stock A, mixed and the Stock A chloroform solution evaporated under a stream of dry nitrogen gas. The dry lipid film was resuspended in water, quantitatively transferred to a volumetric flask (5 mL) and made to volume with water. Stock aqueous solutions of buffer (sodium phosphate monobasic monohydrate; 28.4 mM) and salt (sodium chloride; 105.9 mM) were prepared in water.

**Table 2:** Compositional values of the 8 points, centre point and FaSSIFv1.

Samples	Bile Salt (mM)	Phospholipid (mM)	FFA (mM)	Cholesterol (mM)	pH
1	1.06	0.16	1.04	0.01	6.64
2	11.45	2.48	2.88	0.38	7.12
3	3.4	0.33	2.88	0.09	8.04
4	3.56	1.18	1.04	0.06	5.72
5	3.62	1.25	3.43	0.03	7.14
6	3.35	0.31	0.87	0.17	6.62
7	5.33	0.4	2.96	0.07	6.42
8	2.27	0.96	1.01	0.08	7.34
Centre point (9)	3.46	0.52	1.64	0.032	6.54
FaSSIFv1	3	0.75	1.64	–	6.5

Values from [20], FaSSIFv1 from [14].

##### 2.2.1.2 Fasted state simulated intestinal fluid (FaSSIF)

Pre-prepared media, purchased from Biorelevant.com, were used as described by the manufacturer.

#### 2.2.2 Equilibrium solubility measurement

The method is based on previous papers [14] aliquots (267 µL) of the lipid, buffer and salt stock solutions, an excess of the solid drug and water (3.199 mL) were added Into a centrifuge tube (15 mL Corning® tubes) to make a final aqueous system volume of 4 mL. The pH was adjusted to the required value (Table 1) using 1 M KOH or HCl as required. FaSSIF medium (4 mL) was added to the tube along with an excess of the solid drug and pH was adjusted if required. Tubes were capped and placed into a shaker (Labinco L 28 Orbital shaker) for 1 h at room temperature and the final pH was re-adjusted as required. Tubes were then placed in the shaker at 37 °C for 24 h. Post incubation, an aliquot (1 mL) of each tube was transferred to a 1.5 mL Eppendorf tube and then centrifuged for 15 min, 10,000 rpm. The supernatant was analysed by HPLC for drug content. For each drug, this process was repeated three times and the average value is used.

#### 2.2.3 HPLC analysis

Analysis was performed on a Shimadzu Prominence-i LC-2030C HPLC system using a gradient method for all the drugs. Mobile phases A 10 mM ammonium formate pH 3 (adjusted with formic acid) in water, and mobile phase B 10 mM ammonium formate pH 3 (adjusted with formic acid) in acetonitrile:water (9:1), flow rate 1 mL/min (except acyclovir 0.5 mL/min), time start 70:30 (A:B), 3 min 0:100, 4 min 0:100, 4.5 min 70:30 total run time 8 min. The following columns were used (all at 30 °C): Xbridge® C18 5 µm (2.1 × 50 mm) for ibuprofen, mefenamic acid and griseofulvin, Speck and Burke, ODS-H optimal 5 µm (30 × 150 mm) for acyclovir, furosemide and dipyridamole, and Kromasil 60-5-SIL (3 mm, 15 cm) for paracetamol, The retention time, detection wavelengths and injection volume for each drug are provided in Table 3. For each drug, a concentration curve was prepared using five or six standards that bracketed all the measurement concentrations. For all drugs, the correlation coefficient of the calibration curve was >0.99.

**Table 3:** HPLC conditions.

Drug	Injection volume (µL)	Wave-length (nm)	Retention time (min)
Ibuprofen	100	254	2
Mefenamic acid	10	291	2.3
Furosemide	10	291	2.5
Dipyridamole	10	291	2.5
Griseofulvin	10	291	1.5
Paracetamol	10	254	1.07
Acyclovir	10	254	1.52
Zafirlukast	25	254	2.6
Felodipine	10	254	2.4

#### 2.2.4 Data analysis

Data analysis and comparison was conducted using Graphpad Prism 9 for MacOSX.

## 3 Results and discussion

### 3.1 Equilibrium solubility measurements

**Fig. 1:**
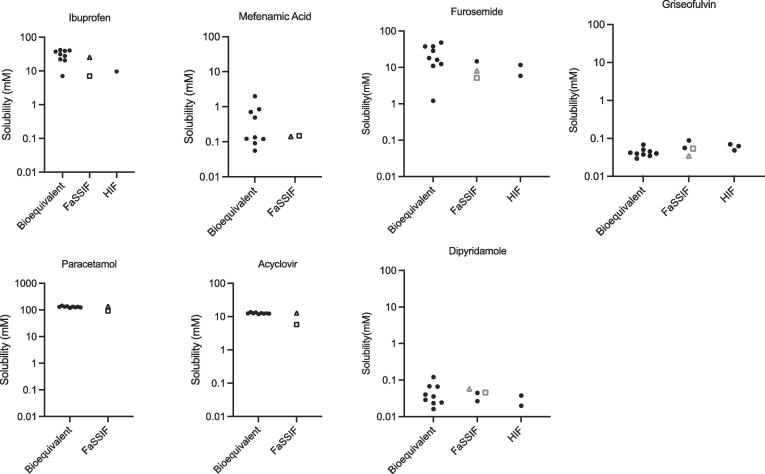
Measured Equilibrium Solubility Distributions. Bioequivalent (mean, n = 3) this study; FaSSIFv1 (Fasted Simulated Intestinal Fluidv1) Δthis study (mean n = 3); □ from [7]) ● from [4]; HIF (Fasted Human Intestinal Fluid) data from [4]. NB Paracetamol y-axis different scale.

The equilibrium solubility results using the nine point bioequivalent media recipes and FaSSIFv1 for the drugs analysed are presented in Fig. 1. The drugs in Fig. 1 (with the exception of griseofulvin) have not previously been analysed in DoE guided fasted media solubility experiments [14], [17], [19], [22] and therefore no comparison with these data sets is possible. Griseofulvin has been previously analysed in the aforementioned DoE guided solubility experiments and comparisons to these data sets are analysed in a previous publication [21]. Where available, literature solubility measurement values for either fasted HIF or SIF are included in Fig. 1. The drugs display solubility behaviour that is consistent with the drug’s physicochemical properties (Table 1) and the solubility drivers identified in the DoE studies [14], [17], [19], [22], see Section 3.3. In addition, for all drugs the majority (for exceptions see next paragraph) of published fasted HIF or SIF solubility values sit within the solubility range measured using the nine bioequivalent media recipes, indicating that the measured bioequivalent solubility envelope is consistent with the available fasted HIF or fasted SIF data.

Three drugs (griseofulvin, paracetamol and acyclovir) present a different behaviour with a very narrow solubility range (solubility multiplier <4, Fig. 1 and Table 4) in the bioequivalent media recipes, indicating that their solubility is not greatly influenced by media composition variation. This narrow solubility range behaviour was present for griseofulvin, as well as for tadalafil and phenytoin, in the initial statistically guided study [14] and has been replicated in a recent study that re-examined the behaviour of the original twelve drugs in the bioequivalent media system [21]. This result adds a further two drugs to this behaviour list (paracetamol and acyclovir), indicating that the multi-point solubility analysis is revealing an intestinal solubility property or behaviour that a single point measurement would miss. For these drugs, three points out of eleven literature solubility values for either fasted HIF or SIF sit outside the bioequivalent solubility range, indicating a level of variability between values. However, there are variations in the measurement protocols applied and based on the very narrow solubility range for the drugs this difference is probably related to the measurement protocols.

**Table 4:** Equilibrium solubility data and analysis.

Drug	Dose (mg)	Estimated Human Peff (cms^−1^ × 10^-4^)	FaSSIFv1 Solubility (mg ml^−1^)	Centre Point Solubility (mg ml^−1^)	Minimum Solubility (mg ml^−1^)	Maximum Solubility (mg ml^−1^)	Solubility Multiplier^1^	Skew^2^
Ibuprofen	400*	12*	5.26	4.27	1.46	6.44	4.41	0.772
Mefenamic Acid	250*	14*	0.0341	0.0289	0.0134	0.481	35.9	29.2
Furosemide	80*	0.6*	4.84	4.12	0.398	15.9	40.0	3.16
Dipyridamole	100*	1.5*	0.0133	0.0145	0.00813	0.0608	7.48	7.23
Paracetamol	500*	1.3*	20.6	19.9	18.0	22.0	1.22	1.10
Griseofulvin	500*	8.7*	0.0122	0.0133	0.0104	0.0240	2.32	3.63
Acyclovir	800*	0.25*	2.86	2.87	2.67	3.07	1.15	0.929

* Data from Butler [7] #9838; ^**^Data from Martindale Extra Pharmacopoeia.

1 Solubility Multiplier = (Maximum Solubility)/(Minimum Solubility).

2 Skew = ((Maximum Solubility − Centre Point Solubility))/((Centre Point Solubility − Minimum Solubility)).

This narrow solubility range behaviour is not restricted to a single BCS/DCS class (paracetamol – class I; griseofulvin – class II; acyclovir – class III) and is probably related to drug molecular structure and properties since these three compounds are relatively simple planar molecules with a low logP value (Table 1). This consistent solubility behaviour, irrespective of media composition might be interesting to examine in relation to possible biopharmaceutical performance implications and biowaivers. Further experimental studies will be required to confirm and fully elucidate this interesting observation and maybe link to drug structure and properties.

### 3.2 Solubility range

**Fig. 2:**
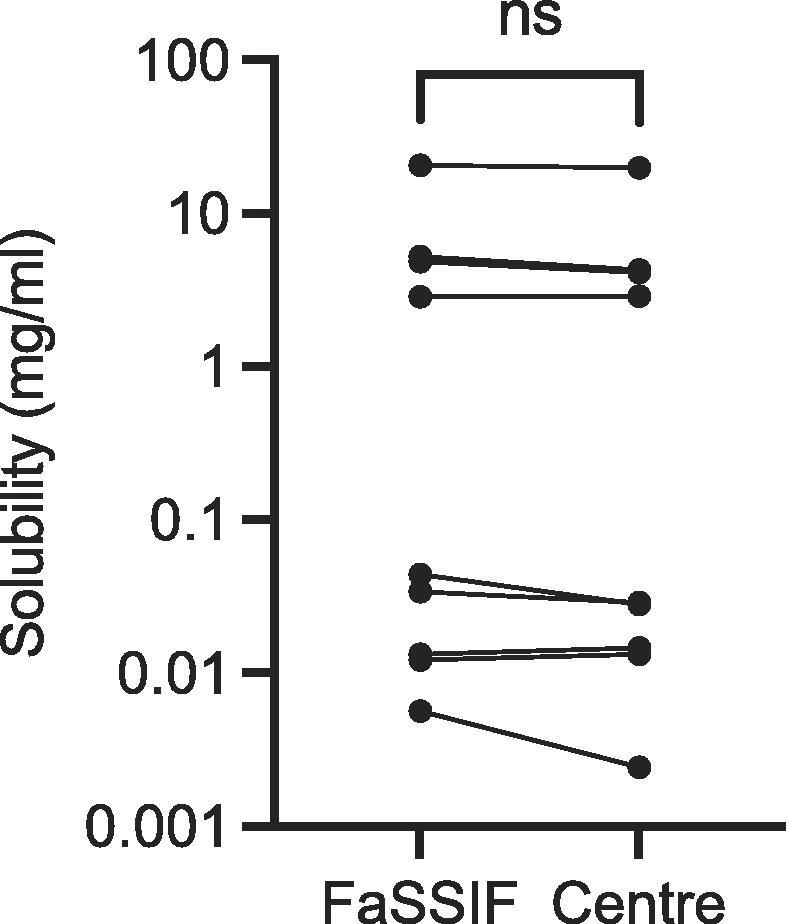
FaSSIFv1 vs Centre Point Solubility Comparison. FaSSIFv1 (Fasted State Simulated Intestinal Fluid) and Centre this study. ns no significant difference (P > 0.05), each point mean n = 3.

Collected solubility data are presented in Table 4, along with data from the original Developability Classification System paper [7]. A statistical comparison of the calculated mean FaSSIFv1 and centre point solubility values (Fig. 2) indicates that there is no statistically significant difference between the two data sets using a Wilcoxon matched pairs signed rank test. Individual drug based non-parametric statistical comparisons of FaSSIFv1 vs centre point measurements (n = 3 per drug for both systems) does not detect a statistically significant difference between the values (P < 0.05) for any drug, results not shown. This indicates that existing FaSSIFv1 results for these drugs could be utilised as a direct comparison to centre point solubility values measured using the bioequivalent system. However, due to the small number of drugs tested, the inherent spread between the values and utilisation of a non-parametric ranking based comparison, it would be prudent to check this relationship either as further results become available or through multiple measurements of individual drugs.

Using the measured bioequivalent maximum and minimum solubility values, a solubility multiplier can be calculated. The values range from 1.15 for acyclovir to 40 for furosemide and reflect the visual point distributions already presented in Fig. 1. The multiplier’s magnitude is smaller than in the original fasted DoE study [14] where for some drugs a three log variation was detected and this reflects the smaller variation noted in the bioequivalent media system [21] study. This was attributed to the elimination of statistically driven and non-biorelevant combinations of high and low media component concentrations. Using the centre point it is possible to calculate a skew value to determine distribution symmetry, with values ranging from 0.772 to 29.2. Generally, the drugs with the lowest solubility multiplier also have the lowest skew value, however, furosemide deviates from this trend having the largest solubility multiplier with a low skew value. This variation indicates the individualistic drug behaviour in these complicated media systems [23], [24] and further results and discussion with respect to this issue are in the next section. This is the first experimental study that permits the calculation of these values and a greater number of examples is required to assess the utility of this information. At this stage it could be surmised that for drugs with a low solubility multiplier and skew value in vivo bioavailability variability will not be influenced by intestinal solubility variability and other factors permeability and/or metabolism will be more important. For high solubility multiplier and skew drugs intestinal solubility variability along with permeability and/or metabolism will contribute to in vivo bioavailability variability.

Based on these results, and Section 3.1 above, the bioequivalent media system is detecting a relevant solubility range and this range is dependent upon the drug’s physicochemical properties, molecular structure and media composition.

### 3.3 Developability classification system range

**Fig. 3:**
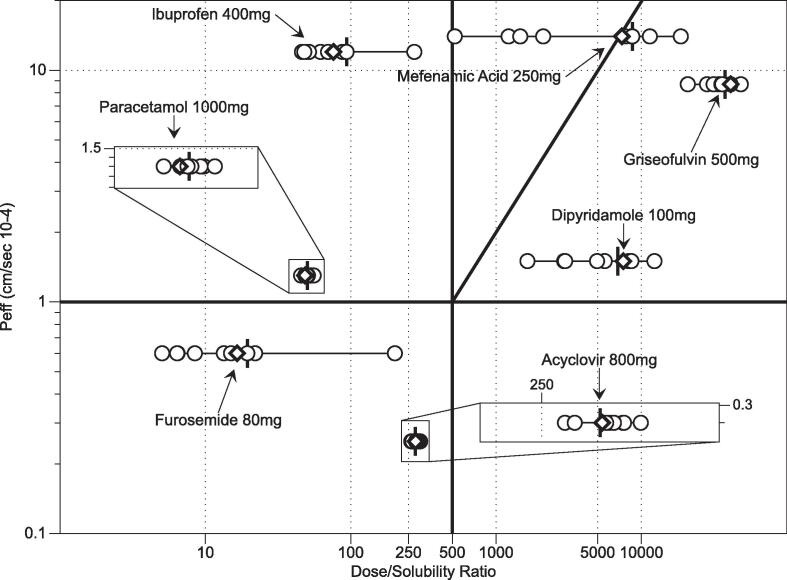
Bioequivalent Systems on Developability Classification System Grid. ♢FaSSIFv1 (Fasted State Simulated Intestinal Fluid); ○ Bioequivalent data points, I Bioequivalent centre point. Inset expanded scale for acyclovir and paracetamol. Individual drugs and doses as labelled. Each point mean n = 3.

**Fig. 4:**
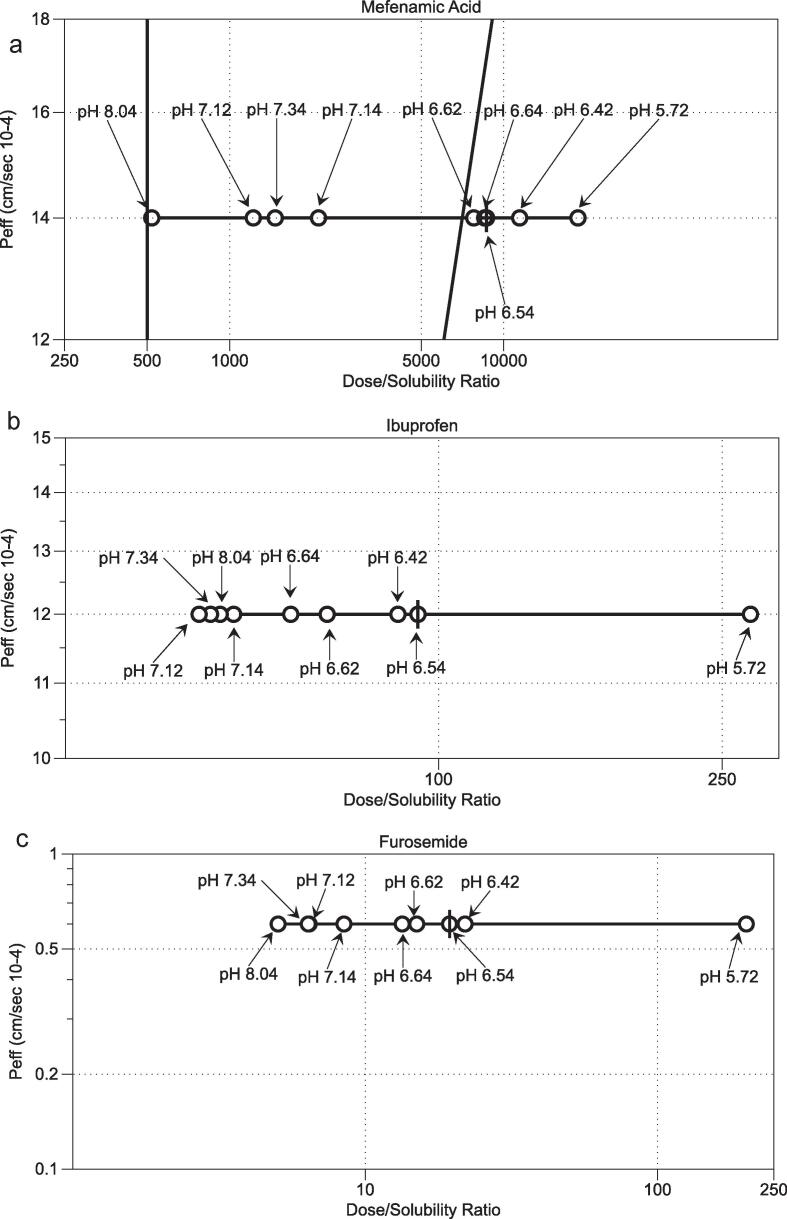
Acidic Drugs pH solubility behaviour. (a) Mefenamic Acid. (b) Ibuprofen. (c) Furosemide. ○ Bioequivalent data points, I Bioequivalent centre point. Measurement pH values as labelled. Each point mean n = 3.

Using the drug’s human intestinal permeability values along with the normal oral dosages (Table 4) [7], it is possible to plot the results on the Developability Classification System grid by calculating a dose/solubility ratio for each measurement point. This is presented in Fig. 3 using FaSSIFv1 and the nine bioequivalent media system measurements. The plot highlights the solubility range along with the multiplier and distribution issues discussed above. The behaviour of the acidic drugs, mefenamic acid, ibuprofen and furosemide with respect to measurement pH is illustrated in Fig. 4a–c. A predominant effect is that solubility increases (therefore dose/solubility volume decreases) with increasing pH with minor variation due to the amphiphilic factors present in the media. This is consistent with the solubility drivers identified for acidic drugs in the original fasted DoE study [14] and other related studies [17], [19], [22]. This indicates that although the media component concentrations and ratios have been changed to provide equivalence to the measured HIF samples [20], the system’s solubility behaviour remains consistent with previous DoE studies.

The bioequivalent point compositions describe greater than ninety percent of the compositional variation present in the analysed HIF samples [20] from twenty, healthy young adult (18–31 years) volunteers [11]. Therefore, it is reasonable to assume that the measured range for each drug in Fig. 3 represents greater than ninety percent of a drug’s solubility behaviour in the measured fasted intestinal space and the calculated maximum and minimum values indicate a drug’s intestinal fasted solubility range. The lowest solubility or largest dose/solubility volume ratio could be taken to represent a worst case scenario with greater than ninety percent of the distribution above this extreme limit, exhibiting a higher solubility. Therefore compound screening or formulation selection based on the lowest solubility point rather than a centre point or average fasted SIF point might be useful as a worst case for a cautious risk based quality by design approach [8]. In addition, this eliminates the inherent risk associated with solubility range distributions if a centre point or average fasted SIF value is utilised, without any knowledge of the solubility range.

For the drugs presented in Fig. 3 only mefenamic acid crosses a DCS boundary from IIb solubility limited to IIa dissolution limited and of note is that the centre point and FaSSIFv1 value is located at or close to the boundary. The additional range based information arising from the multi-point measurement indicates that a worst case formulation approach for mefenamic acid should be based on solubility limited performance rather than dissolution approaches. This demonstrates the utility of using a range over a single value measured either in fasted HIF or SIF. Investigation of more drugs will reveal further candidates where different aspects of these scenarios are likely to arise.

By applying the biopharmaceutical calculations detailed in the introduction a solubility limited absorbable dose (SLAD) and a target particle size to avoid dissolution rate limiting issues [7], [8] can be calculated. The calculation has been applied to the measured centre point and lowest solubility value as a worst case situation (Table 5), using Peff values for each drug from the literature [7] and standard values for other properties. A comparison between the outputs arising from the centre point and lowest solubility measurements exhibit the same relationship described above and in 3.1, 3.2. For the narrow solubility distribution drugs (paracetamol, acyclovir and griseofulvin) there is minimal difference between the values, whilst for the other drugs the difference reflects the discussion above. This hints that a narrow intestinal solubility range might be a useful drug development target, since the drug would then be intrinsically resistant to intestinal solubility variability. The authors recognise this might be an unrealistic target based on current medicinal chemistry structures. For four drugs (paracetamol, ibuprofen, furosemide and acyclovir), the calculated lowest SLAD is above the administered dose (Table 4, Table 5) and therefore minimal solubility based absorption issues are possible, reflective of their positions on the BCS/DCS grid. For three drugs (mefenamic acid, dipyridamole, and griseofulvin), the calculated lowest SLAD is below the dose (Table 4, Table 5) and therefore the lowest solubility based calculation could be applied as a quality by design parameter for particle size to reduce the risk of absorption issues [8].

**Table 5:** Calculated biopharmaceutical data.

Drug	SLAD (mg)		Particle Radius (µm)	
	Centre Point Solubility	Minimum Solubility	Centre Point Solubility	Minimum Solubility
Ibuprofen	24,519	8380	253	148
Mefenamic Acid	193	90	20	14
Furosemide	1181	114	248	77
Dipyridamole	10	6	15	11
Paracetamol	12,357	11,183	545	519
Griseofulvin	55	43	14	12
Acyclovir	3434	3186	207	200

Solubility Limited Absorbable Dose – SLAD = S_INT_ × V × A_n_ where S_INT_ is the intestinal solubility (mg/ml) measurement as indicated in column header (see Table 3), V is the volume of intestinal fluid (500 mL) and A_n_ is the absorption number ($A_{n}=\frac{P_{\mathrm{eff}}\times T_{\mathrm{si}}}{R}$) where P_eff_ is the effective permeability of the intestine to the drug (see Table 3), T_si_ is the small intestinal transit time (3.32 h) and R is the intestinal radius (1.25 cm).

Particle radius = $\sqrt{3D\times S_{\mathrm{INT}}\times T_{\mathrm{si}}/D_{n}\times \rho }$ where D is the diffusion coefficient (typically at 5 × 10^−6^ cms^−1^), S_INT_ and T_si_ are as above, D_n_ is the dissolution number (set to 1) and ρ is the drug density (typically 1.2 g cm^−3^).

### 3.4 Fasted solubility distributions

**Fig. 5:**
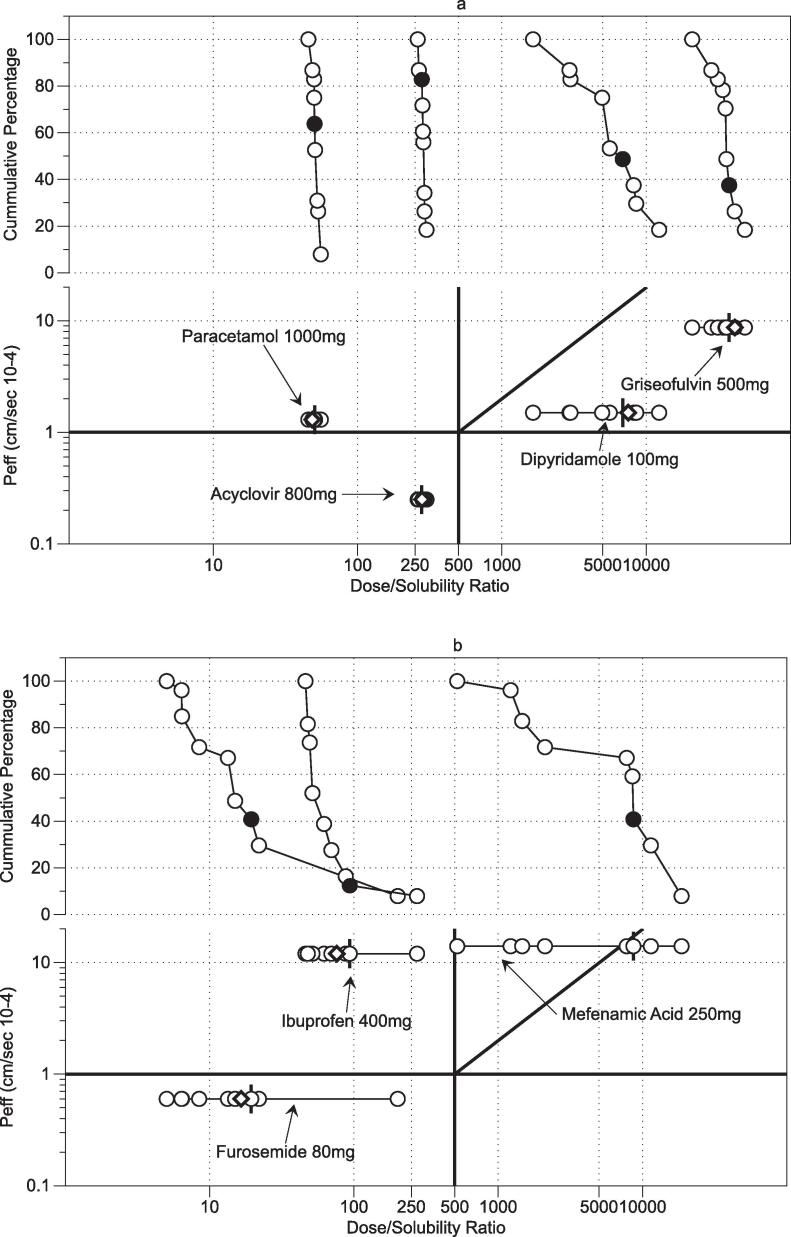
Cumulative dose/solubility ratio distributions. Lower graph: Developability Classification System Grid, ♢ FaSSIFv1 (Fasted State Simulated Intestinal Fluid); ○ Bioequivalent data points, I Bioequivalent centre point. Each point mean n = 3. Upper graph: Cumulative percentage incidence of HIF data points, ○ Bioequivalent data points, ● Bioequivalent centre point.

The bioequivalent point compositions (Table 2) were calculated to describe the compositional variation present in the 152 fasted HIF samples within the analysed data set [20]. Through the application of 5-dimensional Euclidean space it is possible to calculate the proximity of each data set point to an individual bioequivalent point composition to produce a frequency distribution based on the number of data set points closest to each bioequivalent point. Since the study has measured the equilibrium solubility of each bioequivalent point, this can then be converted to a dose/solubility volume frequency distribution, see Fig. 5a and b. It should be noted that this frequency distribution arises from the sampled fasted HIF point compositions [11], [20] and cannot be related to measured in vivo pharmacokinetic variability [25] at this stage. NB Drugs split between figures on basis of presentation clarity.

In Fig. 5a the distributions for paracetamol, acyclovir, griseofulvin and dipyridamole are presented. Based on the presentation in Fig. 1 and associated discussion in Section 3.1, paracetamol, acyclovir and, griseofulvin all have very narrow frequency distributions with almost vertical cumulative lines, related to the very narrow solubility range for these drugs. The points are not evenly distributed on the cumulative plot and only for paracetamol does the centre point occur in the middle of the distribution. Dipyridamole has a broader distribution range but the points are not evenly distributed on the cumulative plot and centre point is towards the lower end of the cumulative plot. In Fig. 5b the distributions for mefenamic acid, ibuprofen and furosemide are presented. Since these are all acidic drugs [26] the distributions will be predominantly controlled by pH (see Section 3.3 and Fig. 4a–c), but also display the same characteristics previously described with points not evenly distributed and centre point towards the lower end of the cumulative plot. Mefenamic acid and furosemide also exhibit an increased degree of structure in the cumulative plot with steps indicative of peaks in the distribution.

Statistical analysis of the distributions either for normal or log normal behaviour did not produce significant results. Previous statistical analysis of fasted SIF DoE solubility distributions [17], [19] highlighted that the distributions were not normal, also the fasted HIF data points used to calculate the bioequivalent points [20] were not normally distributed. This result might reflect the well known variability of these fluids [27], [28] and the measurement of solubility in them [10], [15], [24]. Within this bioequivalent system, and presumably HIF as well, the traverse from low to high solubility points is not a simple vector based on a single concentration of a media component, where a solubilisation relationship might be expected [29], [30], but a five dimensional [23] (and in HIF more) transit through a complex compositional space. Therefore, the lack of an organised statistical distribution when traversing the solubility range based on individual discrete points is to be expected. This might represent an evolutionary aspect to HIF providing variability that maximises nutrient solubilisation, but also impacts administered drugs. This highlights why a single HIF aspirate will not be representative of the entire HIF space and single measurements limited by a lack of knowledge of the sample’s position in the space, which will be further complicated when drug properties are superimposed. This makes prediction difficult and points that knowledge of the solubility distribution via measurement is required with the information potentially useful for performing, as discussed, a risk analysis for the likely impact of solubility variability on absorption behaviour.

### 3.5 Solubility limited absorbable dose distribution

**Fig. 6:**
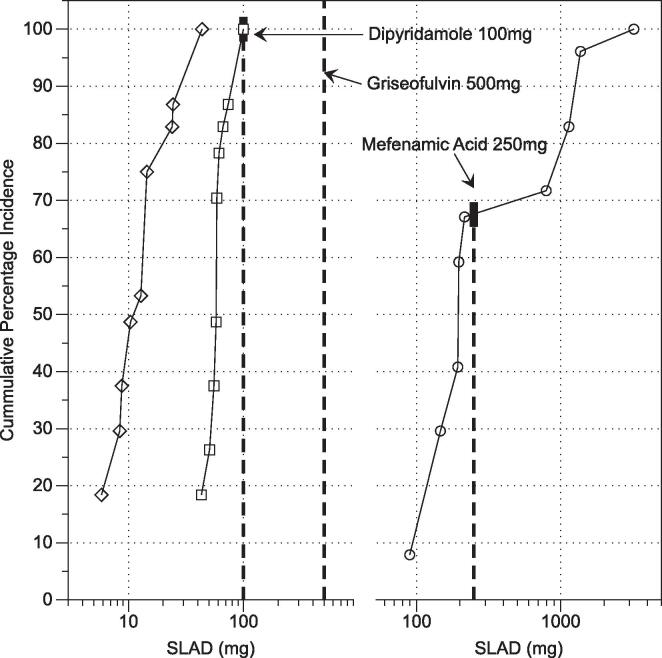
Cumulative Percentage Incidence of Solubility Limited Absorbable Dose. ○ Mefenamic Acid; ♢ Griseofulvin; □ Dipyridamole. Vertical line drug dose, value as indicated.

In Section 3.3 a calculated SLAD based on the centre point and an extreme worst case scenario based on the lowest solubility indicated that for mefenamic acid, dipyridamole and griseofulvin solubility and dissolution rate limiting issues are likely to occur upon oral administration. For mefenamic acid (weak acid) and dipyridamole (weak base) [26], modifications could be applied to account for pH changes during transit through the gastric compartment and down the intestinal tract [31], [32]. Investigation of intestinal tract pH indicates that this source of variation in the upper tract diminishes as material transits down the tract. Since griseofulvin is not ionisable, a pH based adaptation is not applicable. However, for mefenamic acid even the centre point (see Table 5) calculation highlights a solubility issue with respect to the dose. By calculating the SLAD values for all bioequivalent points and linking to the cumulative percentage incidence (see Section 3.4), it is feasible to determine where solubility limitations no longer apply. This is presented in Fig. 6 for mefenamic acid, dipyridamole and griseofulvin. Dipyridamole and griseofulvin plots do not reach the required oral dose value of 100 mg or 500 mg respectively and will not be discussed further. For mefenamic acid the plot indicates that solubility limitations will only be resolved in approximately thirty percent of fasted HIF compositions (vertical line Fig. 6) and this information could be applied for a risk assessment based approach to development and formulation. This represents a further advantage of solubility range knowledge and frequency distribution within the range to asses solubility associated biopharmaceutical issues, especially where the drug crosses a classification boundary. As above investigation of more drugs will reveal further candidates where this scenario is likely to arise.

## 4 Conclusions

The results in this paper indicate that the nine bioequivalent media recipes are simple to apply and provide solubility measurements in agreement with literature fasted HIF and SIF values and drug solubility behaviour in agreement with previous DoE studies. Three drugs exhibit a very narrow solubility range that has been revealed by the multi-point analysis and which has not been previously picked up using a single point measurement. This might represent an interesting behaviour category for further biopharmaceutical consideration. Application of the dose/solubility calculation to the bioequivalent points allows a DCS range to be plotted, which represents greater than ninety percent of drug’s intestinal solubility based on the derivation of bioequivalent points. The calculated range provides greater information than single point measurements and the lowest solubility value represents a worst case scenario that could be applied to quality by design approaches during drug screening, development and formulation. The bioequivalent points can be linked to the original HIF data set to provide a frequency distribution for the measured solubility value. The solubility distributions do not follow a normal or log normal pattern, which it can now be concluded is in part due to the measurement points being distributed in multidimensional space. Therefore, the traverse from low to high solubility points is not a simple vector based on a single concentration or property. In addition, the distribution can be used to refine quality by design risk assessments since it provides a population value for solubility behaviour. Overall the results indicate that the small scale fasted bioequivalent study provides greater information than single point measurements in either fasted HIF or SIF, by determining a fasted intestinal solubility range, with a population frequency distribution (based on the original population [11] and analysis [20]) that can be applied to biopharmaceutical calculations and quality by design approaches. The approach is therefore worthy of further development and research to expand the number of drugs analysed, refine the compositional calculations for the bioequivalent points and link in vitro solubility to in vivo pharmacokinetics.

## Declaration of Competing Interest

The authors declare that they have no known competing financial interests or personal relationships that could have appeared to influence the work reported in this paper.

## References

[1] Dressman J.B., Amidon G.L., Fleisher D. (1985). Absorption potential – estimating the fraction absorbed for orally-administered compounds. J. Pharm. Sci..

[2] Amidon G.L., Lennernas H., Shah V.P., Crison J.R. (1995). A theoretical basis for a biopharmaceutic drug classification – the correlation of in-vitro drug product dissolution and in-vivo bioavailability. Pharm. Res..

[3] Curatolo W. (1998). Physical chemical properties of oral drug candidates in the discovery and exploratory development settings. Pharm. Sci. Technol. Today.

[4] Sun D.X., Yu L.X., Hussain M.A., Wall D.A., Smith R.L., Amidon G.L. (2004). In vitro testing of drug absorption for drug ‘developability’ assessment: Forming an interface between in vitro preclinical data and clinical outcome. Curr. Opin. Drug Discov. Devel..

[5] Dressman J.B., Vertzoni M., Goumas K., Reppas C. (2007). Estimating drug solubility in the gastrointestinal tract. Adv. Drug Deliv. Rev..

[6] Yu L.X., Lipka E., Crison J.R., Amidon G.L. (1996). Transport approaches to the biopharmaceutical design of oral drug delivery systems: Prediction of intestinal absorption. Adv. Drug Deliv. Rev..

[7] Butler J.M., Dressman J.B. (2010). The developability classification system: Application of biopharmaceutics concepts to formulation development. J. Pharm. Sci..

[8] Rosenberger J., Butler J., Dressman J. (2018). A refined developability classification system. J. Pharm. Sci..

[9] Augustijns P., Wuyts B., Hens B., Annaert P., Butler J., Brouwers J. (2014). A review of drug solubility in human intestinal fluids: Implications for the prediction of oral absorption. Eur. J. Pharm. Sci..

[10] de la Cruz-Moreno M.P., Montejo C., Aguilar-Ros A., Dewe W., Beck B., Stappaerts J., Tack J., Augustijns P. (2017). Exploring drug solubility in fasted human intestinal fluid aspirates: Impact of inter-individual variability, sampling site and dilution. Int. J. Pharm..

[11] Riethorst D., Mols R., Duchateau G., Tack J., Brouwers J., Augustijns P. (2016). Characterization of human duodenal fluids in fasted and fed state conditions. J. Pharm. Sci..

[12] Bergström C.A.S., Holm R., Jørgensen S.A., Andersson S.B.E., Artursson P., Beato S., Borde A., Box K., Brewster M., Dressman J., Feng K.-I., Halbert G., Kostewicz E., McAllister M., Muenster U., Thinnes J., Taylor R., Mullertz A. (2014). Early pharmaceutical profiling to predict oral drug absorption: current status and unmet needs. Eur. J. Pharm. Sci..

[13] Bou-Chacra N., Melo K.J.C., Morales I.A.C., Stippler E.S., Kesisoglou F., Yazdanian M., Lobenberg R. (2017). Evolution of choice of solubility and dissolution media after two decades of biopharmaceutical classification system. AAPS J..

[14] Khadra I., Zhou Z., Dunn C., Wilson C.G., Halbert G. (2015). Statistical investigation of simulated intestinal fluid composition on the equilibrium solubility of biopharmaceutics classification system class II drugs. Eur. J. Pharm. Sci..

[15] Clarysse S., Brouwers J., Tack J., Annaert P., Augustijns P. (2011). Intestinal drug solubility estimation based on simulated intestinal fluids: comparison with solubility in human intestinal fluids. Eur. J. Pharm. Sci..

[16] Fuchs A., Leigh M., Kloefer B., Dressman J.B. (2015). Advances in the design of fasted state simulating intestinal fluids: FaSSIF-V3. Eur. J. Pharm. Biopharm..

[17] Ainousah B.E., Perrier J., Dunn C., Khadra I., Wilson C.G., Halbert G. (2017). Dual level statistical investigation of equilibrium solubility in simulated fasted and fed intestinal fluid. Mol. Pharm..

[18] Madsen C.M., Feng K.-I., Leithead A., Canfield N., Jørgensen S.A., Müllertz A., Rades T. (2018). Effect of composition of simulated intestinal media on the solubility of poorly soluble compounds investigated by design of experiments. Eur. J. Pharm. Sci..

[19] Perrier J., Zhou Z., Dunn C., Khadra I., Wilson C.G., Halbert G. (2018). Statistical investigation of the full concentration range of fasted and fed simulated intestinal fluid on the equilibrium solubility of oral drugs. Eur. J. Pharm. Sci..

[20] Pyper K., Brouwers J., Augustijns P., Khadra I., Dunn C., Wilson C.G., Halbert G.W. (2020). Multidimensional analysis of human intestinal fluid composition. Eur. J. Pharm. Biopharm..

[21] Abuhassan Q., Khadra I., Pyper K., Halbert G.W. (2021). Small scale in vitro method to determine a bioequivalent equilibrium solubility range for fasted human intestinal fluid. Eur. J. Pharm. Biopharm..

[22] McPherson S., Perrier J., Dunn C., Khadra I., Davidson S., Ainousah B.E., Wilson C.G., Halbert G. (2020). Small scale design of experiment investigation of equilibrium solubility in simulated fasted and fed intestinal fluid. Eur. J. Pharm. Biopharm..

[23] Dunn C., Perrier J., Khadra I., Wilson C.G., Halbert G.W. (2019). Topography of simulated intestinal equilibrium solubility. Mol. Pharm..

[24] Zhou Z., Dunn C., Khadra I., Wilson C.G., Halbert G.W. (2017). Influence of physiological gastrointestinal surfactant ratio on the equilibrium solubility of BCS class II drugs investigated using a four component mixture design. Mol. Pharm..

[25] Sugihara M., Takeuchi S., Sugita M., Higaki K., Kataoka M., Yamashita S. (2015). Analysis of intra- and intersubject variability in oral drug absorption in human bioequivalence studies of 113 generic products. Mol. Pharm..

[26] Teague S., Valko K. (2017). How to identify and eliminate compounds with a risk of high clinical dose during the early phase of lead optimisation in drug discovery. Eur. J. Pharm. Sci..

[27] Elvang P.A., Jacobsen A.C., Bauer-Brandl A., Stein P.C., Brandl M. (2018). Co-existing colloidal phases in artificial intestinal fluids assessed by AF4/MALLS and DLS: A systematic study into cholate & (lyso-) phospholipid blends, incorporating celecoxib as a model drug. Eur. J. Pharm. Sci..

[28] Elvang P.A., Bohsen M.S., Stein P.C., Bauer-Brandl A., Riethorst D., Brouwers J., Augustijns P., Brandl M. (2019). Co-existing colloidal phases of human duodenal aspirates: Intraindividual fluctuations and interindividual variability in relation to molecular composition. J. Pharm. Biomed. Anal..

[29] Naylor L.J., Bakatselou V., Dressman J.B. (1993). Comparison of the mechanism of dissolution of hydrocortisone in simple and mixed micelle systems. Pharm. Res..

[30] Pedersen B.L., Mullertz A., Brondsted H., Kristensen H.G. (2000). A comparison of the solubility of danazol in human and simulated gastrointestinal fluids. Pharm. Res..

[31] Koziolek M., Grimm M., Becker D., Iordanov V., Zou H., Shimizu J., Wanke C., Garbacz G., Weitschies W. (2015). Investigation of pH and temperature profiles in the GI tract of fasted human subjects using the Intellicap((R)) system. J. Pharm. Sci..

[32] Tsume Y., Mudie D.M., Langguth P., Amidon G.E., Amidon G.L. (2014). The biopharmaceutics classification system: Subclasses for in vivo predictive dissolution (IPD) methodology and IVIVC. Eur. J. Pharm. Sci..

